# A Specific Nuclear DNA Background Is Required for High Frequency Lymphoma Development in Transmitochondrial Mice with G13997A mtDNA

**DOI:** 10.1371/journal.pone.0118561

**Published:** 2015-03-04

**Authors:** Osamu Hashizume, Haruka Yamanashi, Makoto M. Taketo, Kazuto Nakada, Jun-Ichi Hayashi

**Affiliations:** 1 Faculty of Life and Environmental Sciences, University of Tsukuba, 1-1-1 Tennodai, Tsukuba, Ibaraki, Japan; 2 Graduate School of Life and Environmental Sciences, University of Tsukuba, 1-1-1 Tennodai, Tsukuba, Ibaraki, Japan; 3 Department of Pharmacology, Graduate School of Medicine, Kyoto University, Yoshida-Konoe-cho, Sakyou-ku, Kyoto, Japan; 4 International Institute for Integrative Sleep Medicine (WPI-IIIS), University of Tsukuba, 1-1-1 Tennodai, Tsukuba, Ibaraki, Japan; 5 TARA center, University of Tsukuba, 1-1-1 Tennodai, Tsukuba, Ibaraki, Japan; RIKEN Advanced Science Institute, JAPAN

## Abstract

We previously found that mouse mitochondrial DNA (mtDNA) with a G13997A mutation (G13997A mtDNA) controls not only the transformation of cultured lung carcinoma cells from poorly metastatic into highly metastatic cells, but also the transformation of lymphocytes into lymphomas in living C57BL/6 (B6) mice. Because the nuclear genetic background of the B6 strain makes the strain prone to develop lymphomas, here we examined whether G13997A mtDNA independently induces lymphoma development even in mice with the nuclear genetic background of the A/J strain, which is not prone to develop lymphomas. Our results showed that the B6 nuclear genetic background is required for frequent lymphoma development in mice with G13997A mtDNA. Moreover, G13997A mtDNA in mice did not enhance the malignant transformation of lung adenomas into adenocarcinomas or that of hepatocellular carcinomas from poorly metastatic into highly metastatic carcinomas. Therefore, G13997A mtDNA enhances the frequency of lymphoma development under the abnormalities in the B6 nuclear genome, and does not independently control tumor development and tumor progression.

## Introduction

Mitochondrial respiration defects and the resultant enhanced glycolysis under normoxia, that is, the Warburg effect, enable cell growth under hypoxia, and thus are thought to be involved in tumor development [1–4]. Because pathogenic mtDNA mutations also induce mitochondrial respiration defects and the Warburg effect, age-associated accumulation of pathogenic mutations in mtDNA and the resultant age-associated expression of mitochondrial respiration defects are considered to be responsible for tumor development [5, 6]. In fact, somatic mutations are preferentially accumulated in tumor mtDNA [7–9].

In contrast, our previous studies provided convincing evidence that mtDNA with a pathogenic G13997A mutation in the ND6 gene (G13997A mtDNA) controls the malignant transformation of carcinoma cells from a poorly metastatic phenotype into a highly metastatic one [10], although mtDNA does not control tumor development (the transformation of normal cells into tumor cells) [10, 11]. Moreover, the induction of high metastasis was not due to respiration defects and the resultant Warburg effect, but to overproduction of reactive oxygen species (ROS) [12].

Subsequently, we generated transmitochondrial mito-mice-ND6^13997^ (B6mtND6^13997^) carrying the nuclear genome from B6 mice and G13997A mtDNA from highly metastatic carcinoma cells [13], and showed that they developed lymphoma with high frequency [14], indicating the possible involvement of mtDNA mutations in tumor development. However, no tumor development was observed in transmitochondrial mito-mice-COI^6589^ (B6mtCOI^6589^) with T6589C mtDNA [14]. Because these mice expressed respiration defects and the Warburg effect [15], but did not overproduce ROS, we proposed that ROS overproduction but not the Warburg effect would be responsible for high frequency lymphoma development [14].

These findings raise several questions: Does G13997A mtDNA independently induce lymphomas even in mice with a nuclear genetic background that is not prone to develop lymphomas? Does G13997A mtDNA also induce high metastasis in tumors developed in mice, given that it induces high metastasis in a low metastatic lung carcinoma cell line [10]? To answer these questions, here we generated mice possessing G13997A mtDNA and nuclear genetic background derived from the A/J strain, which is not prone to develop lymphoma [16] and from mice that are prone to develop hepatocellular carcinomas [17, 18]. Moreover, we treated the mice with urethane to enhance lung adenoma development [19, 20], and examined its effects on the malignant transformation of adenomas into adenocarcinomas in mice with G13997A mtDNA.

The results suggest that G13997A mtDNA enhances the frequency of lymphoma development that is primarily caused by abnormalities in the B6 nuclear genome. Moreover, it does not always enhance transformation of normal cells in mice or malignant transformation of tumor cells developed in mice, probably due to the requirement of some nuclear abnormalities.

## Materials and Methods

### Ethics statement

All animal experiments were performed in accordance with protocols approved by the Institutional Animal Care and Use Committee of University of Tsukuba, Japan (Permit Number: 12-264, 13-312, and 14-271).

### Mice

B6 mice were purchased from CLEA Japan (Tokyo, Japan), and A/J mice were purchased from Japan SLC (Shizuoka, Japan). Mito-miceND6^13997^ (B6mtND6^13997^ mice) were generated previously [13]. Lkb1 (+/−) mice were obtained from Kyoto University. Female B6 and B6mtND6^13997^ mice were crossed with B6, A/J and Lkb1 (+/−) males. F_1_ males obtained from the cross between B6 or B6mtND6^13997^ females with A/J males were used for urethane treatment experiments. F_1_ females obtained from the cross between B6 females or B6mtND6^13997^ females with A/J males were furthermore backcrossed to A/J males to obtain F_2_ and F_3_ generations. F_3_ males were used to study the spontaneous lung tumor formation. Mice were monitored every day for general health, and those with signs of tumor burden, such as hunched posture, ruffled coats, and respiratory distress, were euthanized by cervical dislocation. The maximum tumor size (diameter) was less than 7 mm in sacrificed mice. When mice were sacrificed, anesthesia with an intraperitoneal injection of 2.5% avertin was employed to minimize animal suffering. All mice were maintained on hardwood bedding on a 12-h light/dark cycle and given food and water ad libitum.

### Measurement of ROS production in mitochondria

ROS generation was detected with the mitochondrial superoxide indicator MitoSOX-Red (Invitrogen, Carlsbad, CA, USA). 1×10^5^ cells were incubated with 1 mM MitoSOX-Red for 15 min at 37°C in phosphate-buffered saline (PBS), washed twice with PBS, and then immediately analyzed with a FACScan flow cytometer (Becton Dickinson, San Jose, CA, USA). Data were analyzed with FlowJo software (Tree Star, USA). Relative ROS levels were calculated as mean fluorescence intensity of MitoSOX-Red stained cells minus autofluorescence of the unstained cells.

### Urethane treatment

B6 strain mice were injected intraperitoneally (IP) at 4 weeks of age with 1 mg/g body weight urethane (ethyl carbamate; SIGMA, St. Louis, MO, USA) weekly for a total of 10 doses. Groups of F_1_ hybrid mice were injected IP at 4 weeks of age with 1 mg/g body weight urethane weekly for a total of 2 doses. Tumor multiplicities were examined 52 weeks after the initial urethane injection.

### Histological analyses

Lungs and livers were fixed in formalin solution. Tumors greater than 0.5mm in diameter on the lung surface were counted after fixation. After overnight fixation in 10% buffered formalin, tissues were paraffin embedded, cut into 8-μm sections, placed on glass slides, and stained with hematoxylin and eosin. Formalin-fixed, paraffin-embedded serial sections were used for histological analysis.

### Statistical analysis

We analyzed data with the (unpaired or paired) Student t-test. Kaplan−Meier curves were assessed with the log-rank test. Values with P < 0.05 were considered significant.

## Results

### Generation of A/J mice with G13997A mtDNA (A/JmtND6^13997^) by backcrossing

First, we asked whether B6 mtDNA with the G13997A mutation (G13997A mtDNA) in B6mtND6^13997^ mice independently regulates lymphoma development. To answer this question, we needed to generate A/JmtND6^13997^ mice carrying G13997A mtDNA in the nuclear genetic background of the A/J strain, which is not predisposed to developing lymphoma [16].

To generate A/JmtND6^13997^ mice, an F_1_ female obtained by mating a B6mtND6^13997^ female with an A/J male was backcrossed to an A/J male, and the resultant F_2_ females were further backcrossed to A/J males, resulting in the generation of F_3_ mice (A/JmtND6^13997^). A/JmtND6^13997^ mice thus carry a nuclear genetic background derived mostly from the A/J strain and possess only G13997A mtDNA (Table 1). As a negative control, we generated F_3_ A/JmtB6 mice carrying B6 mtDNA without the G13997A mutation with a nuclear genetic background derived mostly from the A/J strain. A/JmtND6^13997^ mice and A/JmtB6 mice thus share the nuclear and mitochondrial genetic backgrounds except that A/JmtND6^13997^ mice carry the G13997A mutation in mtDNA (Table 1).

**Table 1:** Effects of nuclear-background variations and the presence or absence of B6-derived mtDNA with the G13997A mutation on the tumor-related phenotypes of mice.

	Strains				No. of mice with tumors / no. of mice examined				
Mice	nuclear DNA	mtDNA	No. of mice	Urethane treatment*	Lymphoma	Pulmonary nodules (No. of nodules)	Liver carcinomas	Lung metastasis (No. of nodules)	others
B6	B6	B6	35	−	3/35**	0/35**	0/35**	0/35**	0/35**
B6mtND6^13997^	B6	B6 (G13997A)	35	−	16/35**	0/35**	0/35**	0/35**	0/35**
A/JmtB6	A/J	B6	5	−	0/5	4/5 (3.75 ± 0.5)	0/5	0/5	0/5
A/JmtND6^13997^	A/J	B6 (G13997A)	6	−	0/6	5/6 (3.2 ± 1.5)	0/6	0/6	0/6
B6	B6	B6	10	++	0/10***	10/10 (8.0 ± 9.7)	0/10	0/10	0/10
B6mtND6^13997^	B6	B6 (G13997A)	12	++	0/12***	12/12 (7.83 ± 6.5)	0/12	0/12	0/12
B6 × A/J (F_1_)	B6 × A/J	B6	6	+	0/6	6/6 (17.3 ± 3.9)	0/6	0/6	0/6
B6mtND6^13997^ × A/J (F_1_)	B6 × A/J	B6 (G13997A)	5	+	0/5	5/5 (21.6 ± 8.0)	0/5	0/5	0/5
Lkb1 (+/−)	B6 (Lkb1+/−)	B6	19	−	0/19****	0/19	14/19	2/14 (2.5 ± 0.7)	0/19
Lkb1 (+/−) mtND6^13997^	B6 (Lkb1+/−)	B6 (G13997A)	16	−	0/16****	0/16	13/16	2/13 (2 ± 0)	0/16

* B6 mice and B6mtND6^13997^ mice sharing the B6 nuclear genetic background received weekly urethane administration for 10 times (++). In contrast, F_1_ hybrids between B6 females and A/J males and F_1_ hybrids between B6mtND6^13997^ females and A/J males received weekly administration of urethane for only twice (+) due to their higher susceptibility to urethane treatment than mice with B6 nuclear genetics background.

** These results were reported previously (Hashizume et al., 2012).

*** No mice developed lymphoma, since they were sacrificed to detect lung adenoma at 12 months of age.

**** No mice developed lymphoma, since they died due to the Lkb1 mutation before lymphoma development.

### Examination of tumor formation in A/JmtND6^13997^ mice

**Fig 1:**
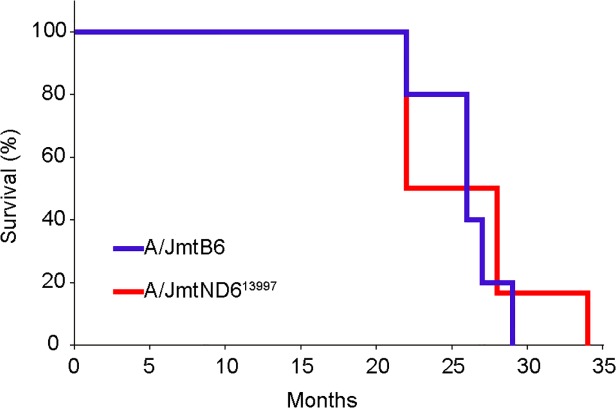
Kaplan-Meier survival curves of A/JmtB6 and A/JmtND6^13997^ mice. Median survival times of A/JmtB6 mice (n = 5) and A/JmtND6^13997^ mice (n = 6) were 25.0 months and 22.0 months, respectively. No statistically significant differences in median survival times were observed (p = 0.2216).

Five A/JmtB6 males and six A/JmtND6^13997^ males were available to investigate the effects of the A/J nuclear genetic background on lifespan and the spectrum of tumor formation. Median survival times of A/JmtB6 and A/JmtND6^13997^ mice were 25.0 months and 22.0 months, respectively (Fig. 1), and were similar to those observed in B6 mice (26.1 months) and B6mtND6^13997^ mice (24.6 months) with the B6 nuclear genetic background [14]. Thus, the lifespans were not affected by the presence or absence of the G13997A mutation or by the difference in the nuclear genetic backgrounds.

Gross necropsy of all euthanized moribund A/JmtB6 mice and A/JmtND6^13997^ mice showed that no mice developed lymphomas or other tumors except pulmonary nodules, irrespective of whether they possessed the G13997A mutation (Table 1). Given that the nuclear genetic background of the A/J strain is not prone to develop lymphoma, these data indicate that the B6 nuclear genetic background, which predisposes mice to develop lymphoma [16, 21–23], is required to induce high frequency lymphoma development in B6mtND6^13997^ mice [14]. Moreover, the G13997A mutation in mtDNA does not independently induce lymphoma development. Thus, G13997A mtDNA simply enhances the lymphoma-prone phenotype that is regulated by the B6 strain-derived nuclear genome. To confirm this idea, larger studies using more animals than were used in this study are required, because we were unable to exclude the possibility that there was a low level of lymphoma formation in F_3_ mice with the A/J nuclear background.

**Fig 2:**
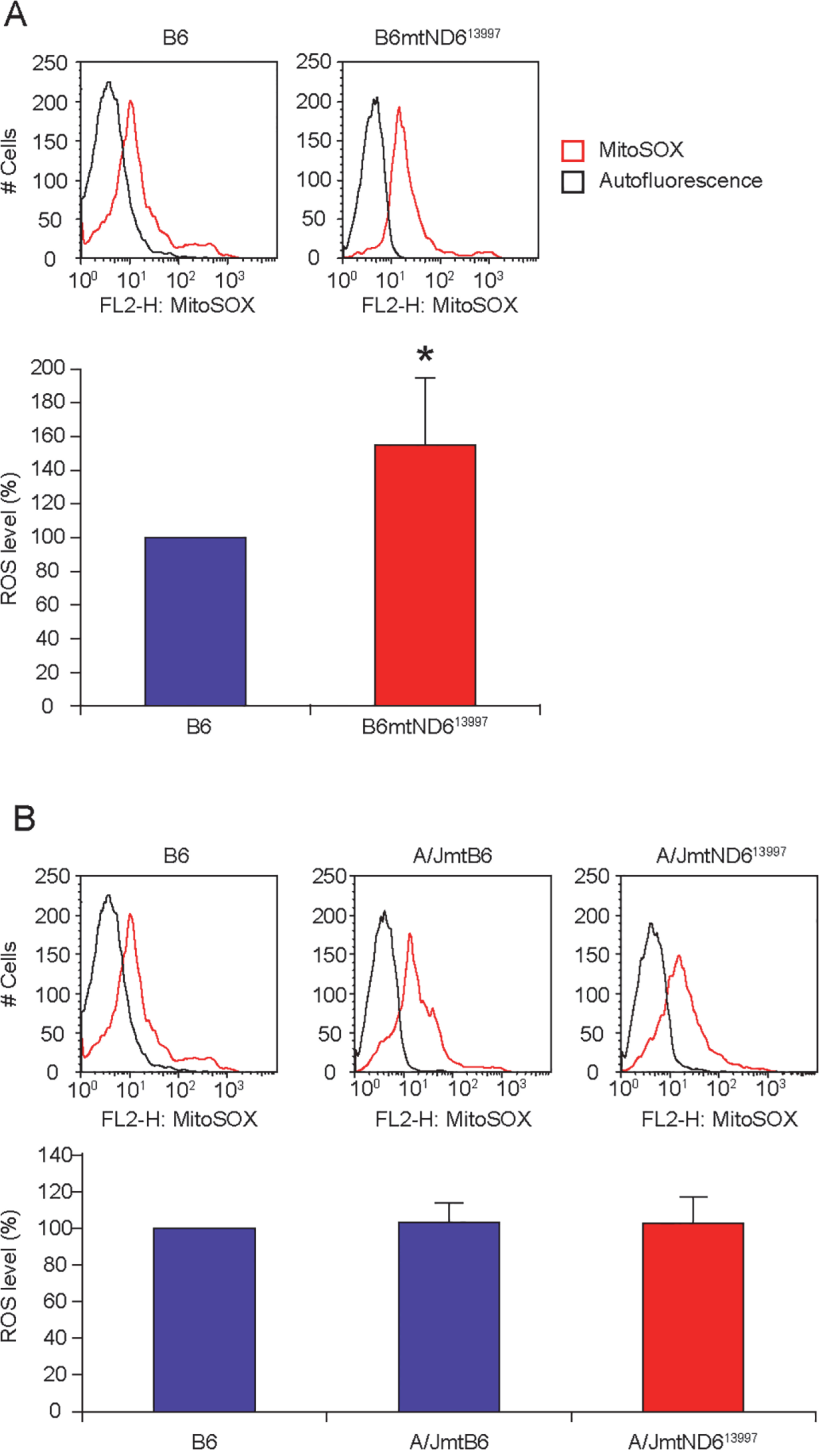
Estimation of mitochondrial superoxide (i.e., reactive oxygen species, ROS) levels in bone marrow cells after treatment with MitoSOX-Red. (A) ROS levels in bone marrow cells from mice with B6 nuclear background. (B) ROS levels in bone marrow cells from mice with A/J nuclear background. B6 mice were used as controls. Upper panels, representative flow cytometry histograms of MitoSOX-Red. Lower panels, relative ROS levels calculated by FlowJO as mean fluorescence intensity for MitoSOX-Red minus background autofluorescence of the unstained cells. Data are presented as mean values with SD (n = 3). *P < 0.05 compared with control B6 mice.

We then compared ROS levels in bone marrow cells of A/JmtB6 (18-month-old male) and A/JmtND6^13997^ (18-month-old male) mice, using age-matched B6 males and B6mtND6^13997^ males as controls (Fig. 2A). A/JmtND6^13997^ mice did not show ROS overproduction (Fig. 2B), indicating that the A/J nuclear background might possess systems to suppress ROS overproduction by G13997A mtDNA. In contrast, ROS levels of B6 mice and A/JmtB6 mice were similar (Fig. 2B), indicating that suppression of lymphoma development in A/J mice but not in B6 mice was due to some nuclear factors in A/J mice that suppress signals of lymphoma development.

**Fig 3:**
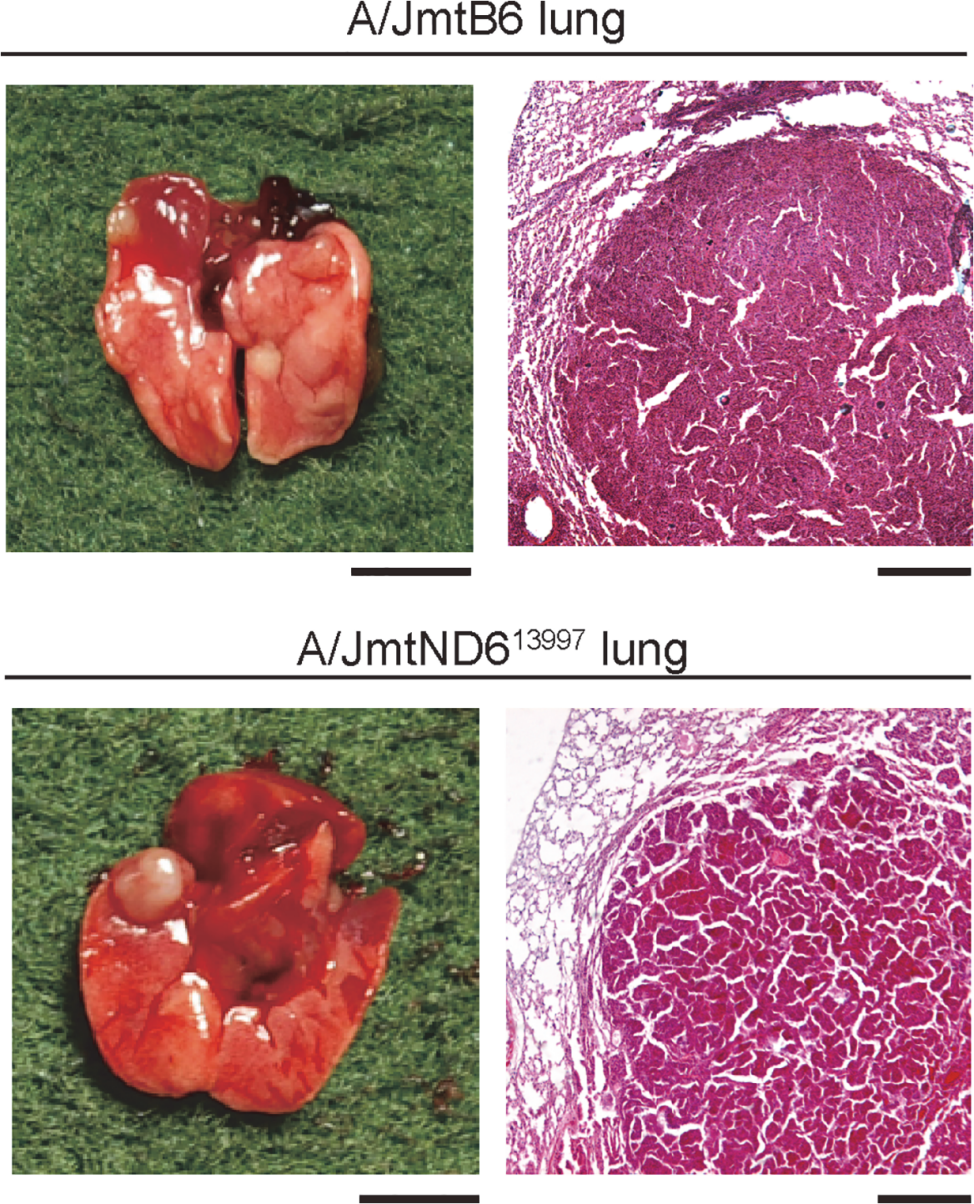
Lung adenoma formation in A/JmtB6 and A/JmtND6^13997^ mice. Gross necropsy (left) and hematoxylin and eosin staining of histological sections (right) of the lung. Most mice with the A/J nuclear genetic background developed lung adenomas. The frequencies of lung adenoma formation in A/JmtB6 and A/JmtND6^13997^ mice were 80% (4/5) and 83.3% (5/6), respectively. Scale bars: left, 5 mm; right, 200 μm.

The mice with the A/J nuclear genetic background frequently developed pulmonary nodules (Table 1). The results were expected, because A/J mice are known to develop lung adenomas [16]. However, the numbers of pulmonary nodules did not differ substantially between A/JmtB6 mice and A/JmtND6^13997^ mice (Table 1). Our histological analysis of the pulmonary nodules suggested that they were all lung adenomas (Fig. 3). These observations indicate that the G13997A mutation in mtDNA does not enhance either the number of lung adenomas or the malignant progression of lung adenomas into lung adenocarcinomas in mice with the A/J nuclear genetic background.

### Effects of G13997A mtDNA on malignant progression of urethane-induced adenomas

To further assess whether G13997A mtDNA enhances the malignant progression of lung adenomas into adenocarcinomas, we treated the mice with urethane to induce early onset and enhance the frequency of lung adenoma development. Because F_1_ hybrids between B6 and A/J mice are more susceptible to lung adenoma development than are B6 mice [19], we used six F_1_ hybrids between B6 females and A/J males and five F_1_ hybrids between B6mtND6^13997^ females and A/J males. We also used ten B6 mice and twelve B6mtND6^13997^ mice.

Based on urethane-administration protocols [19, 20], B6 mice and B6mtND6^13997^ mice, which share the same B6 nuclear genetic background received 10 weekly urethane injections beginning 4 weeks after birth. In contrast, F_1_ hybrids between B6 females and A/J males and F_1_ hybrids between B6mtND6^13997^ females and A/J males received only 2 weekly injections of urethane due to their higher susceptibility to urethane treatment than that of mice with the B6 nuclear genetic background.

The urethane-treated mice were examined one year after the birth. All of the mice developed pulmonary nodules, but the F_1_ hybrids had more pulmonary nodules than did mice with the B6 nuclear genetic background, even though the latter mice received more urethane treatments (Table 1). There was no noteworthy difference in nodule numbers between the B6 and B6mtND6^13997^ mice or between the F_1_ hybrids with or without the G13997A mutation in their mtDNA (Table 1).

**Fig 4:**
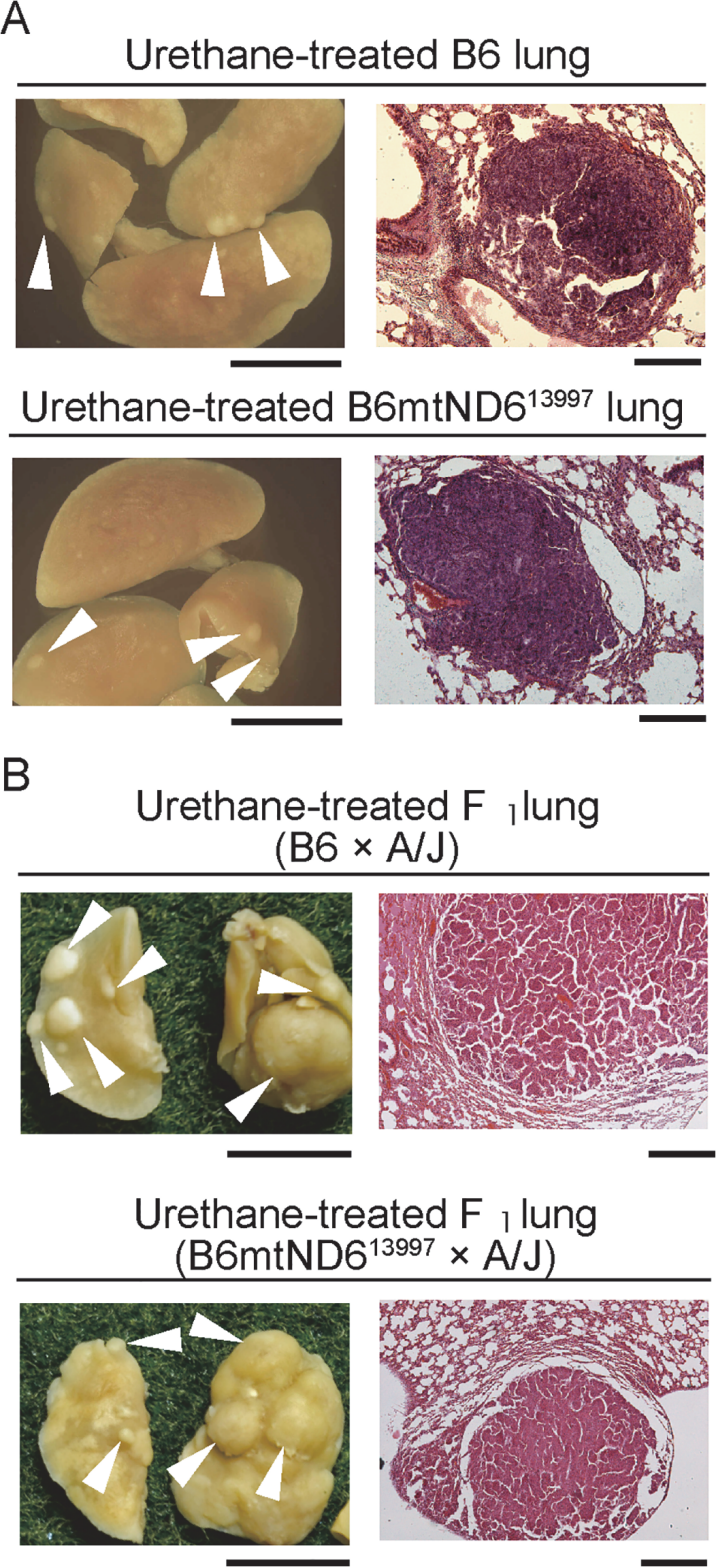
Enhanced development of lung adenomas after urethane administration. Urethane-treated mice with the nuclear genetic background of B6 (A) and of F_1_ hybrids between B6 and A/J mice (B). Gross necropsy of formalin-fixed lungs (left), which were sectioned and stained with hematoxylin and eosin (right). Small tumors (indicated by arrowheads) were observed on the surface of the lungs. Histological analysis showed that these tumor-like abnormalities corresponded to adenomas. The numbers of lung tumors that developed in B6, in B6mtND6^13997^, and in F_1_ hybrids between B6 females × A/J males and between B6mtND6^13997^ females × A/J males were 8.0 ± 9.7, 7.8 ± 6.5, 17.3 ± 3.9 and 21.6 ± 8.0, respectively. Scale bars: (A) left, 5mm; right, 100μm, (B) left, 5mm; right, 200μm.

Histological analysis of the pulmonary nodules (Fig. 4) showed that they were all lung adenomas: malignant progression to adenocarcinomas was not observed even in mice possessing G13997A mtDNA (B6mtND6^13997^ mice and F_1_ hybrids between B6mtND6^13997^ females and A/J males). These results demonstrate that G13997A mtDNA does not induce the tumor progression of lung adenomas into adenocarcinomas, even when the early onset and frequency of adenoma development are enhanced by urethane administration.

### Effects of G13997A mtDNA on the metastasis of hepatocellular carcinomas in Lkb1 (+/−) mice

Finally, we investigated whether G13997A mtDNA enhances metastasis in mice with a nuclear abnormality that induces the development of metastasis-prone carcinomas. To this end, we used Lkb1 (+/−) mice with the B6 nuclear genetic background to introduce G13997A mtDNA. The Lkb1 gene is a tumor suppressor gene that plays a role in cell cycle arrest and apoptosis [17]. Most Lkb1 (+/−) mice develop hepatocellular carcinomas within 40 weeks of birth; 10% of them subsequently form lung metastases in the ensuing 10 weeks [18]. Our previous studies showed that G13997A mtDNA is responsible for the high frequency of lymphoma development in lymphoma-prone B6 mice [14] and for the high frequency of lung metastasis in metastasis-prone lung carcinoma cells [10]. Therefore, we expected that G13997A mtDNA would also induce a high frequency of lung metastasis in metastasis-prone Lkb1 (+/−) mice [18].

**Fig 5:**
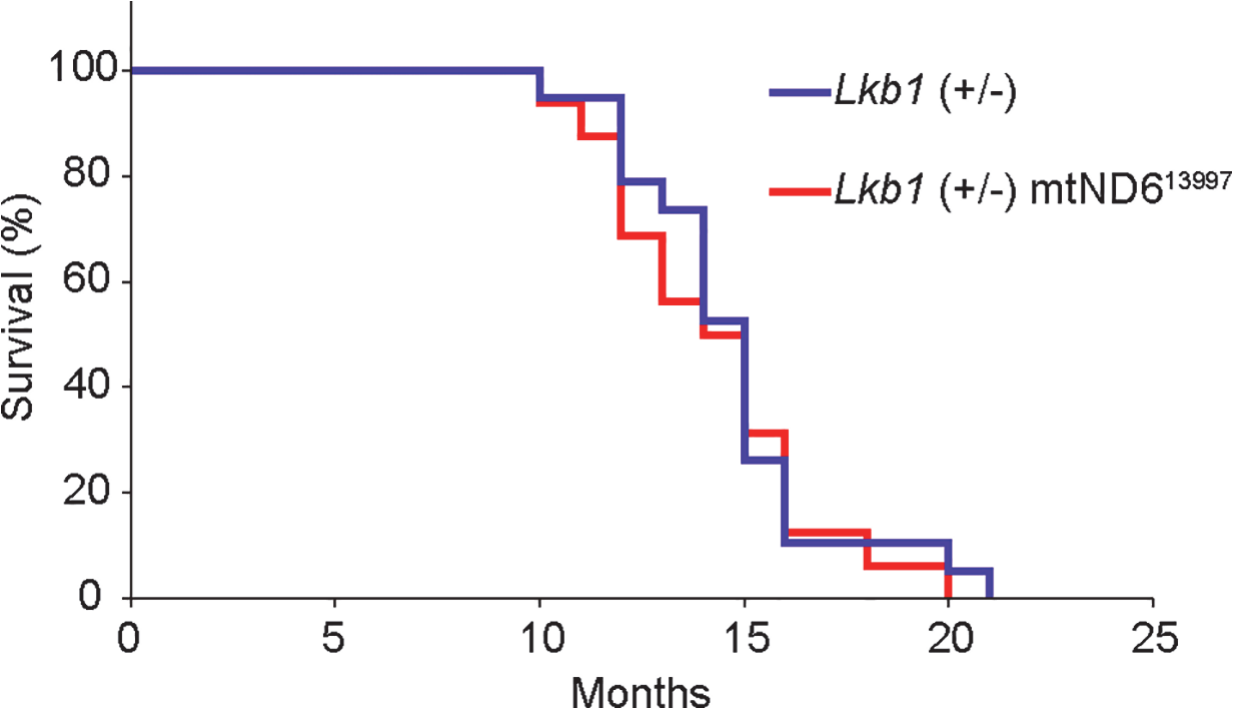
Kaplan-Meier survival curves of Lkb1 (+/−) and Lkb1 (+/−) mtND6^13997^ mice. Median survival times of Lkb1 (+/−) (n = 19) and Lkb1 (+/−) mtND6^13997^ mice (n = 16) were 14.1 months and 14.0 months, respectively. No statistically significant differences in median survival times were observed between them (p = 0.1892).

First, we mated B6mtND6^13997^ females to Lkb1 (+/−) males, and obtained F_1_ Lkb1 (+/−) mice possessing G13997A mtDNA (Lkb1 (+/−) mtND6^13997^). Then, we monitored 19 male Lkb1 (+/−) mice and 16 male Lkb1 (+/−) mtND6^13997^ mice for signs of tumor formation. The median survival times for the Lkb1 (+/−) mice and Lkb1 (+/−) mtND6^13997^ mice were 14.1 and 14.0 months, respectively, and no statistically significant differences were observed between them (Fig. 5).

**Fig 6:**
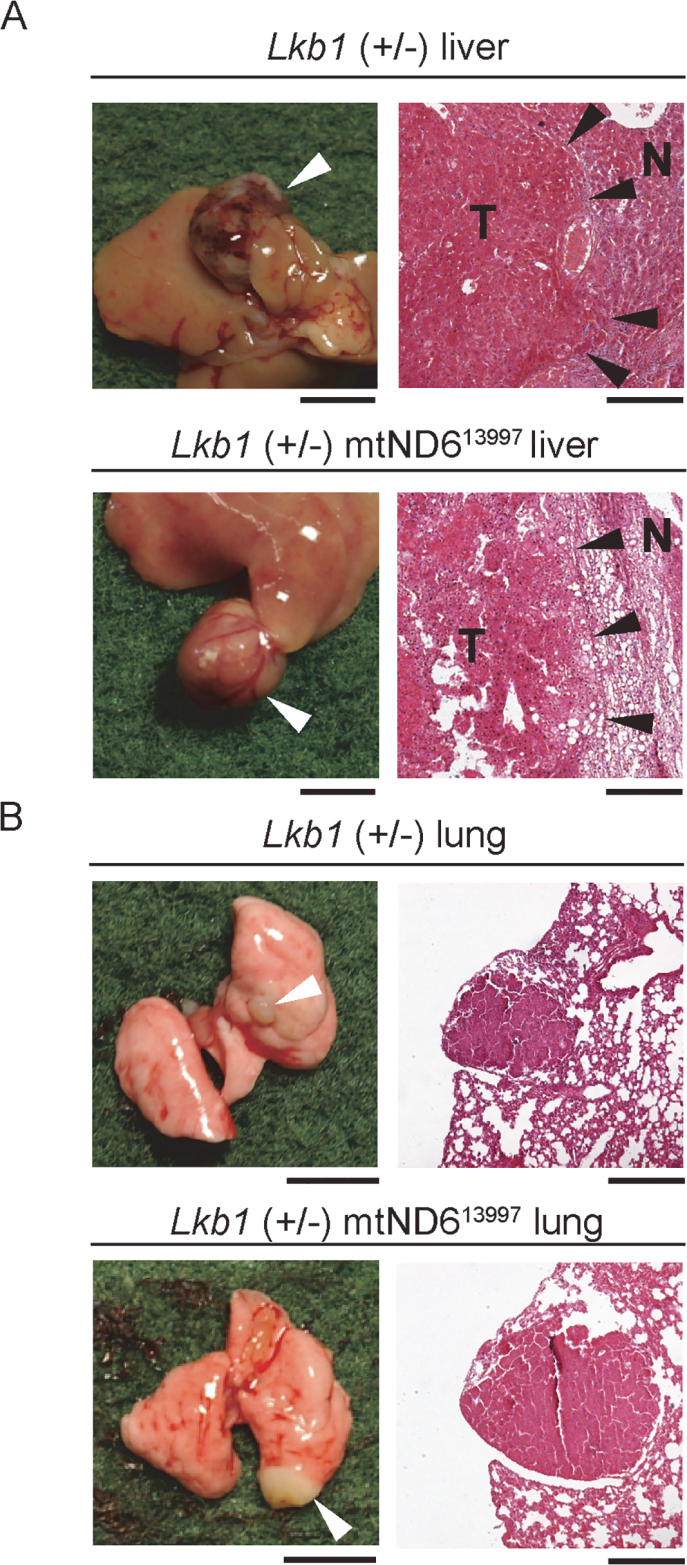
Development of tumors in the liver and metastatic nodules in the lungs of Lkb1 (+/−) and Lkb1 (+/−) mtND6^13997^ mice. Gross necropsy (left) and histological sections (right) of livers (A) and lungs (B). White arrowheads indicate hepatocellular carcinomas in the liver (A) and metastatic nodules in the lung (B). Black arrowheads on the liver sections (A) indicate the borders between the developed tumors (T) and the normal tissues (N). The frequencies of hepatocellular carcinoma formation in Lkb1 (+/−) and Lkb1 (+/−) mtND6^13997^ mice were 73.7% (14/19) and 81.3% (13/16), respectively. The frequencies of lung metastasis in Lkb1 (+/−) and Lkb1 (+/−) mtND6^13997^ mice were 14.3% (2/14) and 15.4% (2/13), respectively. Scale bars: (A) left, 5 mm; right, 100 μm, (B) left, 5mm; right, 200 μm.

Gross necropsy of all of the euthanized moribund mice showed that 14 of the 19 Lkb1 (+/−) mice and 13 of the 16 Lkb1 (+/−) mtND6^13997^ mice developed tumor-like abnormalities in the liver (Table 1). Histological analysis of the liver abnormalities revealed that all of these tumor-like abnormalities were hepatocellular carcinomas (Fig. 6A). Moreover, two of the 14 Lkb1 (+/−) mice and two of the 13 Lkb1 (+/−) mtND6^13997^ mice that developed hepatocellular carcinomas had 2–3 lung nodules (Table 1, Fig. 6B). Therefore, the frequencies of lung metastasis did not change substantially in Lkb1 (+/−) mtND6^13997^ mice, indicating that the G13997A mutation in mtDNA did not enhance lung metastasis in Lkb1 (+/−) mice.

## Discussion

The current study showed that G13997A mtDNA, which enhances lymphoma development through ROS overproduction in B6mtND6^13997^ mice with the B6 nuclear genetic background [14], does not enhance lymphoma development in A/JmtND6^13997^ mice with the A/J nuclear genetic background, which is not prone to lymphoma development (Table 1). Thus, G13997A mtDNA regulates lymphoma development with the help of the B6 nuclear background. At this time, we do not know what factors in B6 mice help the lymphoma development. To identify the nuclear factors that are involved in lymphoma development in B6 mice, it would be helpful to isolate two B6 sublines that show low and high frequency of lymphoma development, respectively, and to compare their whole nuclear genome sequences.

With respect to the malignant transformation of tumors, G13997A mtDNA, which enhanced the lung metastasis of a lung carcinoma cell line [10], neither induced malignant transformation of lung adenomas nor enhanced the lung metastasis of hepatocellular carcinomas (Table 1). Therefore, G13997A mtDNA does not independently enhance transformation of normal cells (tumor development) or malignant transformation of tumor cells, probably due to the requirement of some nuclear abnormalities.

Our previous study [15] revealed that B6mtCOI^6589^ mice, which have the B6 nuclear genetic background and carry homoplasmic T6589C mtDNA in their COI gene, exhibited a low frequency of lymphoma development, probably due to the expression of respiration defects in the absence of ROS overproduction. Therefore, the B6 nuclear genetic background as well as mtDNA mutations that induce ROS overproduction appears to be required for a high frequency of lymphoma development in mice.

It has been proposed that accumulation of pathogenic mtDNA mutations and the resultant Warburg effect enhance cell growth under conditions of hypoxia, and thus are involved in tumor development [1–4]. To further assess the idea, we have to generate additional transmitochondrial mice carrying various nuclear genetic abnormalities or mtDNA with various pathogenic mutations that induce respiration defects and/or ROS overproduction. Recently, we generated transmitochondrial mice (B6mtRNA^Lys7731^) with the B6 nuclear genetic background and high proportions of mtDNA containing the G7731A mutation in their tRNA^Lys^ gene [24]. Because G7731A mtDNA simultaneously induces mitochondrial respiration defects and modest ROS overproduction [24], and ROS overproduction has been linked to heart failure [25], we plan to examine whether these mice will show a high frequency of lymphoma development or heart failure as they age.
